# Absorbable cyst brushes

**DOI:** 10.1007/s10544-023-00674-y

**Published:** 2023-08-23

**Authors:** Filipe Marques, Wouter van der Wijngaart, Niclas Roxhed

**Affiliations:** 1 KTH Royal Institute of Technology, Micro and Nanosystems, Malvinas väg 10, 100 44 Stockholm, Sweden; 2 MedTechLabs, Bioclinicum, Karolinska University Hospital, Solna, Sweden

**Keywords:** Cancer, Cysts, Diagnostics, Cell biopsy, Cell brush, Loop brush, Absorbable materials, Fine Needle Aspiration, Endoscopic Ultrasound

## Abstract

Cytobrushes are used for low-invasive sample collection and screening in multiple diseases, with a significant impact on early detection, prevention, and diagnosis. This study focuses on improving the safety of cell brushing in hard-to-reach locations by exploring brush construction from absorbable materials. We investigated the efficacy of loop brushes made of absorbable suture wires of Chirlac, Chirasorb, Monocryl, PDS II, Vicryl Rapid, Glycolon, and Catgut during their operation in conjunction with fine-needle aspiration in an artificial cyst model. PDS II brushes demonstrated the highest efficiency, while Monocryl and Catgut also provided a significant brushing effect. Efficient brushes portrayed higher flexural rigidity than their counterparts, and their efficiency was inversely proportional to their plastic deformation by the needle. Our results open avenues for safer cell biopsies in hard-to-reach locations by utilizing brushes composed of absorbable materials.

**Supplementary Information:**

The online version contains supplementary material available at 10.1007/s10544-023-00674-y.

## Introduction

Cell brushing is a versatile low-invasive technique for isolating, purifying, or collecting cells. Its most common use is for cervical cancer screening, where cells from the cervix are directly brushed for laboratory analysis. Additionally, cell brushing is used to diagnose or man- age cancer (Alsarraf et al. 2018), infections (Onuma et al. 2020), autoimmune disorders (Singh et al. 2015), or genetic disorders in gastroenterology (Nishino et al. 2018), respiratory medicine (Furuya et al. 2021), or urology (Potretzke et al. 2016).

Cytobrushes and swabs are conventionally used instruments for cell brushing. Bristles and fibers of cytobrushes and swabs, respectively, require finesse during design. If these structures are too harsh to tissue, damage may be taken to an extreme resulting in adverse events such as bleeding. However, if not harsh enough, cells cannot be collected. Upon miniaturization, bristles and fibers may become stiffer and/or have smaller contact areas, posing additional challenges to cell brushing instrument design.

Fine-needle aspiration (FNA) is a minimally-invasive procedure for diagnosing cysts and tumors (Muniraj and Aslanian 2017; Zhu et al. 2017). FNA can be performed percutaneously as a standalone procedure or integrated with ultrasound endoscopy (EUS-FNA) to diagnose hard-to-reach lesions. However, cy- topathologists face challenges in providing a diagnosis due to the low cellularity of cystic samples. This is particularly significant in cysts in the pancreas, breast, salivary glands, and thyroid, where low cellularity in samples may occur in up to 67% of cases (Thornton et al. 2013; de Jong et al. 2011; Łukasiewicz et al. 2017; Staibano et al. 2022).

Cytobrushes in their traditional brush form, i.e., typically consisting of a handle and a block of bristles, have been used for cyst brushing. The EchoBrush (Cook Endoscopy, USA) was once a commercially available brush for cysts during FNA but was discontinued due to an increase in adverse events such as bleeding (during and post-procedure) and acute pancreatitis, occurring at a rate between 4 and 10% of procedures (Muniraj and Aslanian 2018; Lariño-Noia et al. 2018). Currently, instruments for cell brushing of cysts during FNA are not commercially available.

Ensuring safety is crucial when operating in hard-to-reach locations within the human body, such as the respiratory tract, gastrointestinal tract, and urinary tract. Retained Surgical Instruments and Unretrieved Device Fragments are items inadvertently left behind during surgery, resulting from human error or device failure, which is estimated to occur in every 1000 to 18.000 surgeries worldwide (Weprin et al. 2021). However, this frequency is possibly underestimated due to underreporting and the exclusion of "near miss" cases (Weprin et al. 2021). Surgical instruments have been reported to brake in 20% of endoscopy procedures, in a retrospective analysis of 39.817 procedures (Yasuhara et al. 2014). Adverse events related to the analysis were attributed to parts falling off bro- ken instruments due to misuse. Increasing safety motivates this study of brushes constructed from absorbable material.

**Fig. 1:**
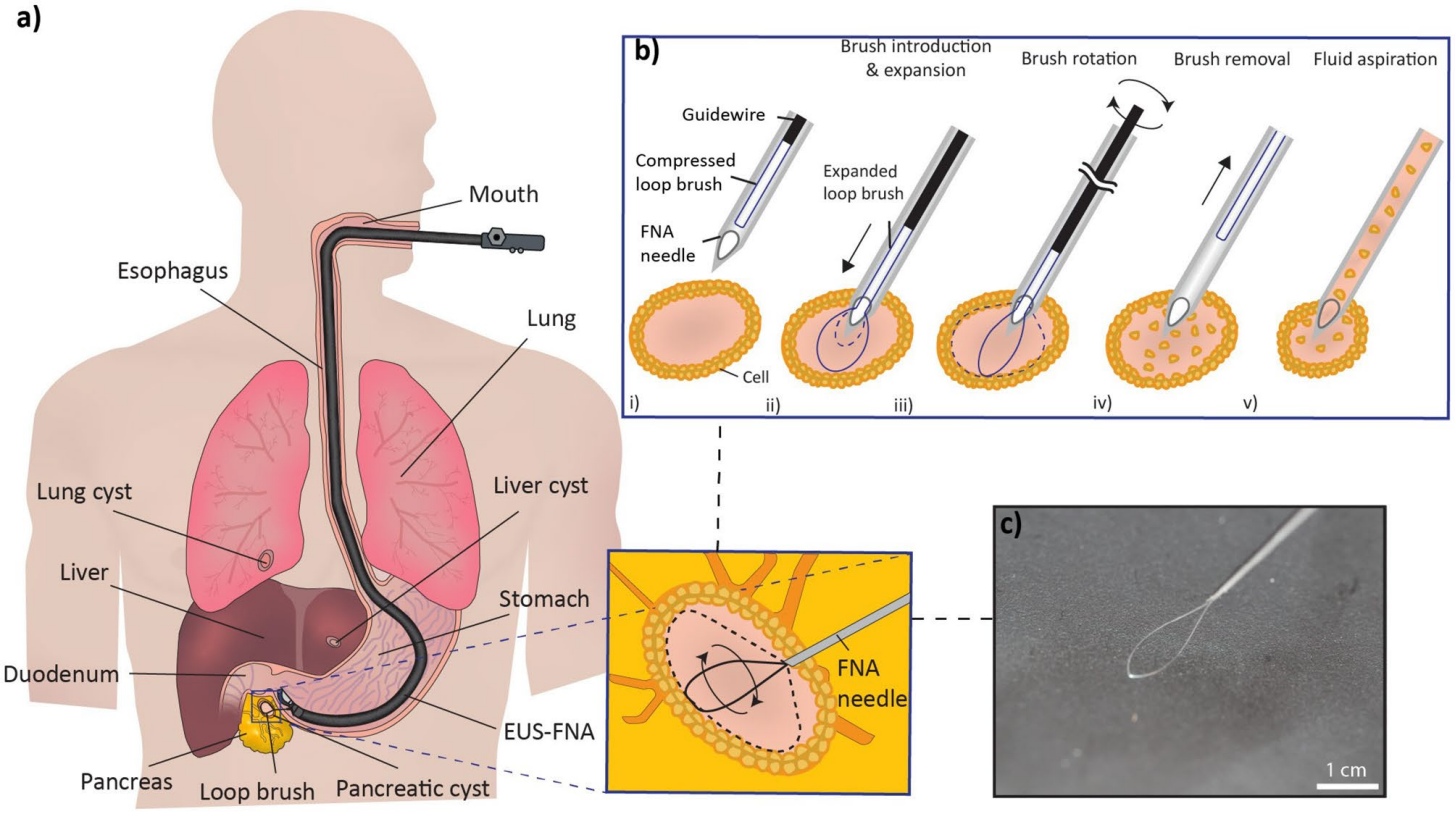
**a** Illustration of an EUS-FNA procedure using a loop brush in a pancreatic cyst. Liver and lung cysts in the illustration depict other potential EUS-FNA targets. **b** Loop brush procedure steps: (i) the loop is compressed within the FNA needle before puncture; (ii) the cyst is punctured, and the loop is introduced into the cyst; (iii) the expanded loop brush is rotated against the inner cyst wall, releasing cells in the liquid of the cyst; (iv) the brush is removed from the needle, and; (v) the liquid is aspirated from the cyst for downstream cytology. **c** Photograph of a PDS II loop brush within a 22G hypodermic needle

Loop brushes have emerged as a solution for cyst brushing during FNA (Marques et al. 2021). These brushes consist of a single loop-shaped wire that conforms to the surrounding environment, being compressed within a thin needle and subsequently expanded to the size of a cyst (Fig. 1). Loop brushes are minimally-invasive while still facilitating the collection of cells from the cysts. This study specifically focuses on loop brushing of cysts in connection with FNA. We investigate the capacity of seven absorbable loop brush materials to collect cells in man-made cysts, given their capacity to be absorbed by the human body.

## Methods

### Loop brushes

**Fig. 2:**
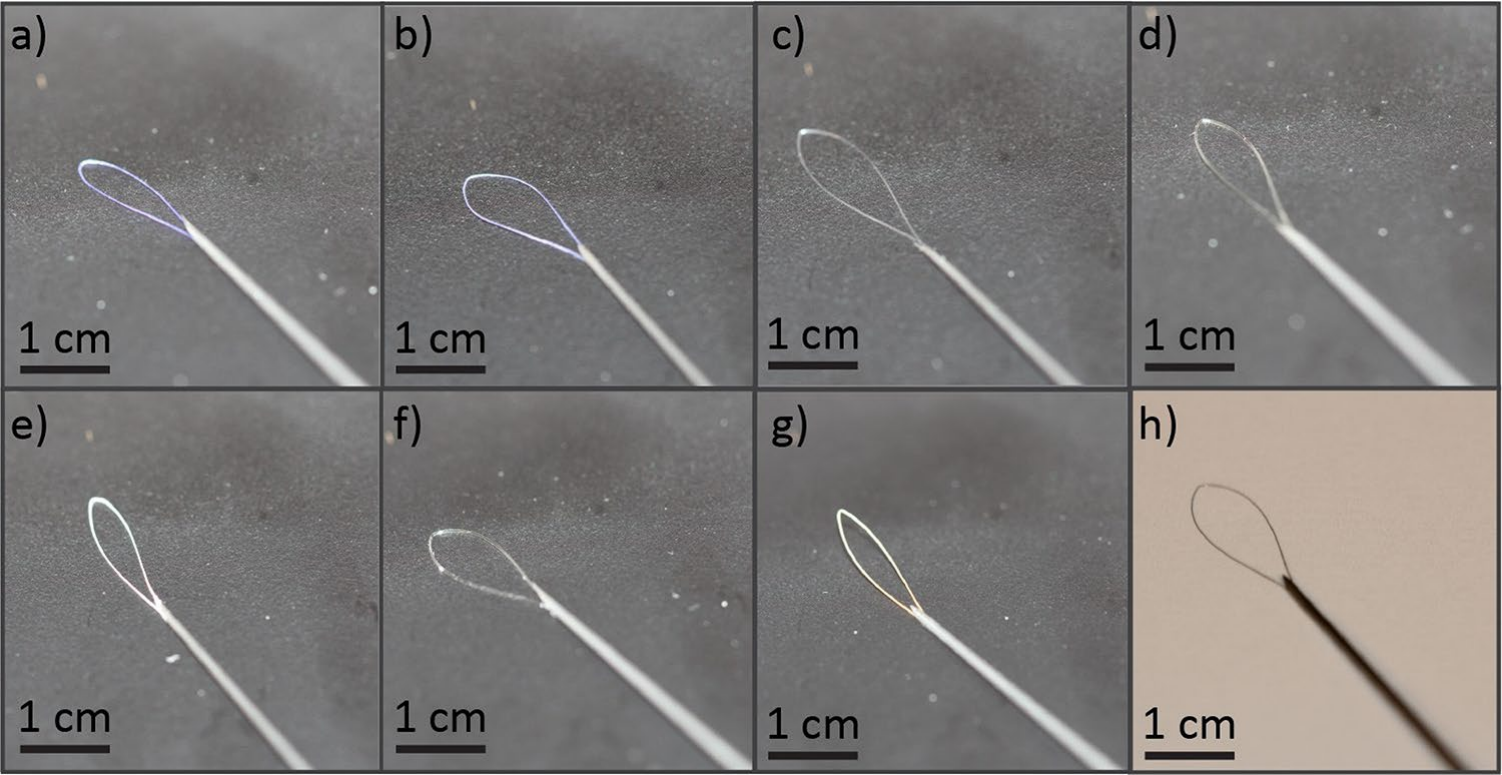
Photographs of loop brushes of different loop materials inserted in 22G needles: **a** Chirlac; **b** Chirasorb; **c** PDS II **d** Monocryl **e** Vicryl Rapid; **f** Glycolon **g** Catgut, and; **h** Nitinol

Loop brushes were designed for operation with a 22G FNA needles and comprise a 1 cm long wire loop attached to a 99 µm nitinol guidewire (Niti#1, straight annealed light oxide, Fort Wayne Metals). The loops are made from one of seven commercially available 6/0 absorbable suture wires: Chirlac (Vitrex, PG0201), Chirasorb (Vitrex, LV0201), Monocryl (Ethicon, W3215), PDS II (Ethicon, Z489E), Vicryl Rapid (Ethicon, W9913), Glycolon (Resorba, PB41504) and Catgut Chrom (SMI, 2,101,512), all obtained from SuturerOnline.se (Malmö, Sweden) (Fig. 2a–h). Given the unique packaging of Catgut in hydrating fluid (isopropanol and water), these sutures were air-dried for 24 h before assembly. Control loop brushes were created using 50 µm diameter nitinol wire (Niti#1, straight annealed light oxide, Fort Wayne Metals) (Marques et al. 2021). Loop brushes were assembled by manually placing wire loop ends adjacent to the guidewire and fixating the parts with 1 µL of 4011 Loctite glue dispensed via a pipette. The loop brushes were then inserted into the lumen of 22G hypodermic needles (4710007040, Henke-Sass Wolf, Germany).

### Cyst model

Pig small intestines were removed immediately post-mortem by Skövde Slakteri AB (Skövde, Sweden) and bagged with a DMEM (10,313,021, Thermo Fisher Scientific, Sweden) based solution with 10% FBS (GibcoTM 10270106, Fisher Scientific, Sweden) and 1% Penicillin- Streptomycin (15,070,063, Thermo Fisher Scientific, Sweden), to prevent tissue decomposition. The organs were refrigerated at 4 °C until use, up to 24 h post-mortem. For cyst preparation, the small intestines were placed on a cutting board and sectioned every 3 cm with a scalpel. Each section was folded to the luminal side, rinsed with tap water for 1 min, and simultane- ously gently cleaned to remove sustenance remnants. Then, intestinal sections were folded back on the apical side, and cable ties (36–7893, Clas Ohlson, Sweden) were positioned and tightened at each end of a section. These intestine sections were placed in a beaker with tap water until cyst filling. Before brushing tests, sections were placed in a petri dish and filled with 10 mL of physiological saline solution using a 27G hypodermic needle (Supplementary Information 1).

### Brushing experiments

**Fig. 3:**
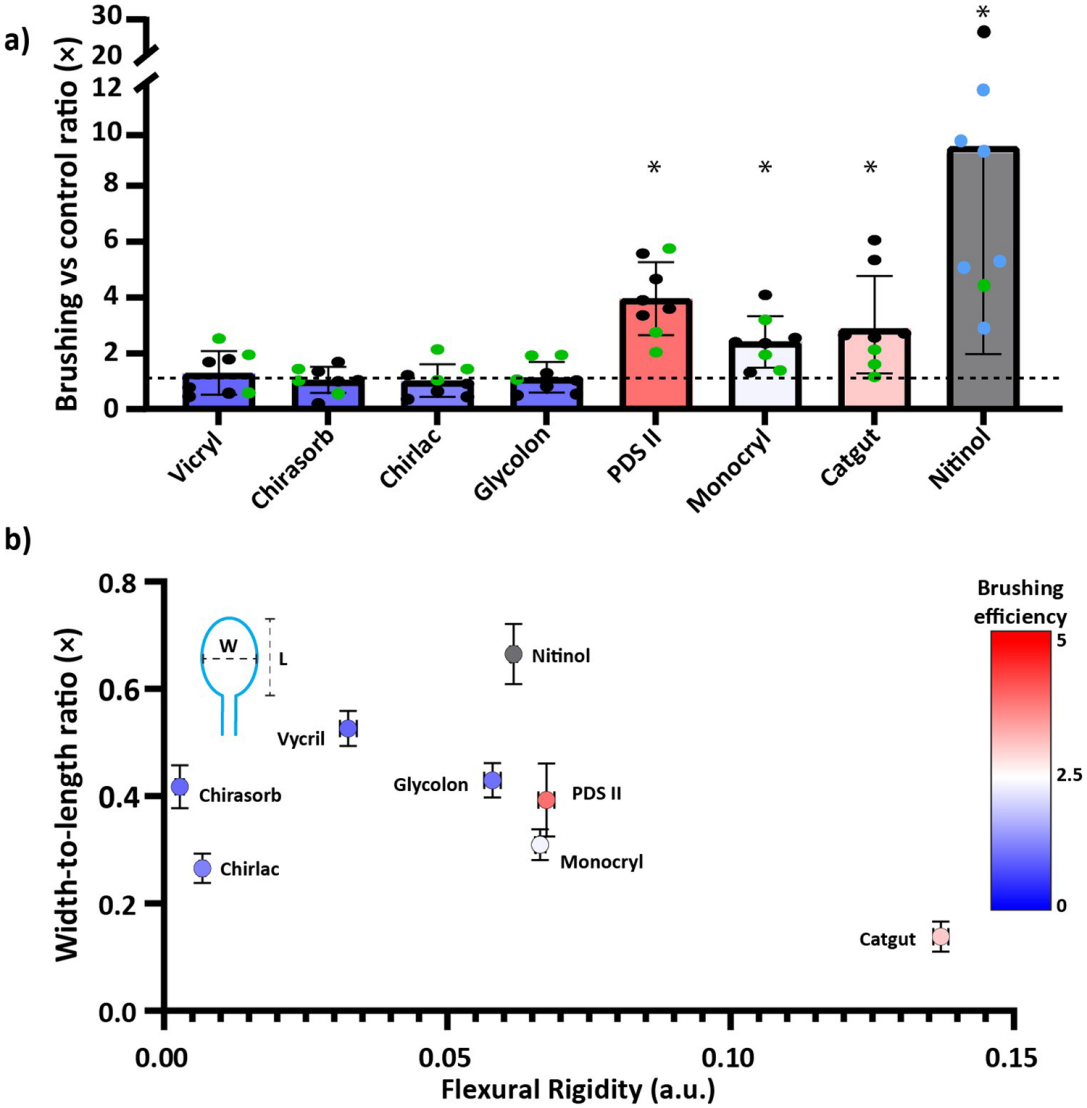
Measured brush efficacies. **a** Ratio of cell counts in FNA sample after brushing vs no brushing (control) for loop brushes of absorbable suture wires of Vicryl Rapid, Chirasorb, Chirlac, Glycolon, Monocryl, PDS II, Catgut and Nitinol. Bar height is mean value; error bars are SD; dashed line is ratio 1 × ; * indicates significant difference between paired controls and brushing (*p*-value = 0.039). **b** Ratio of cell counts between brushing and control (colour) versus the mean flexural rigidity and average width-to-length ratio for loops of absorbable material. Error bars are sd (n = 3)

A total of 58 artificial cysts were prepared, two for positive controls and 56 for brushing. Each cyst was first punctured with a 22G needle and aspirated to obtain a 1.5 mL sample (negative control). Immediately after, another 22G hypodermic needle with an integrated loop brush was used to puncture the cyst. The loop was pushed through the distal end of the guidewire into the cyst and manually rotated three turns clockwise and three turns counter-clockwise for 60 s at an approximate rotation speed of 60 rpm. Then the brush was removed from the cyst, and the needle, and a syringe was inserted into the needle to aspirate 1.5 mL sample. This procedure was repeated for each brushing experiment with two loop brushes of each material. The first and second loop brushes of each material were reused three and five times, respectively (Figure 3a black and green dots). Positive controls were performed with three different brushes (Figure 3a black, green and blue dots). For every experiment, a new cyst was used. Cysts were inspected for perforation after brushing.

### Liquid sample treatment

Liquid samples were transferred into 1.5 mL Eppendorf tubes and subsequently mixed with 500 mL StemProTM AccutaseTM (A1110501, Thermo Fisher Scientific, Sweden), gently homogenized with a pipette by aspirating and dispensing liquid in 2-s cycles for 30 s, and incubated at 37 °C for 5 min. Samples were then filtered using a 70 µm cell strainer (431,751, Corning R, Netherlands) and centrifuged using a IKA™ GL Centrifuge (IKA™, Germany) at 11,000 rpm for 4 min. The supernatant was removed, and a fluorophore solution of saline water with Hoechst 33,342 (H3570, Thermo Fisher Scientific, Sweden), in a 0.05% v/v concentration, was added to each sample. Samples were then mixed with 1.5 mL of staining solution. Finally, the sample tubes were wrapped with aluminum foil for 20 min before cell counting.

### Cell counting

Cell counting was performed with a Countess II FL automated cell counter (Thermo Fisher Scientific, Sweden), coupled with an EVOS™ light cube, DAPI (AMEP4650, Thermo Fisher Scientific, Sweden). All samples were gently homogenized with a pipette, and 10 µL of the sample was placed on a Countess™ cell counting chamber slide (C10228, Thermo Fisher Scientific, Sweden). Cell counting was performed with the following settings on the counter: size between 4–14 µm, brightness between 0–255 a.u., circularity of 0.67, and using autofocus.

Finally, calculations to correct for dilution were performed, taking into account a loss of 1.5 mL from controls to brushing (Supplementary Information 2). Cyst brushing was defined as being efficient for brushing vs. negative control cell count ratios above 1.

### Flexural rigidity measurements

To assess flexural rigidity, a suture wire of each material was inserted into a 22G needle, leaving a 1 cm long section protruding from the distal end. Loctite 4011 was applied to the distal end of the needle to fix the suture to the needle. The needles were positioned vertically with the suture section facing down. A scale (ABS 220-4N, KERN & SOHN, Germany) was placed below the needle, whereafter the needle was moved down with a micrometer positioner such that the lowest position was 0.5 mm below the initial contact point between the suture wire and the scale. During these movements, the suture wire deformed against the scale surface while the induced force, *F*, was recorded (Supplementary Information 3, Table 2). We assumed a displacement distance, *δ*, of 0.5 mm.

For structures with a fixed geometry, the flexural rigidity, *D*, scales as

$$D\cdot \sim \frac{F}{\delta },$$

wherefore the recorded displacement and force values allow a relative comparison of the flexural rigidity among the suture wires.

### Width-to-length measurements

To assess the plastic deformation of the loops by the needles, three loop brushes of each ma- terial (including nitinol) were placed inside 22G hypodermic needles for 24 h, and thereafter protruded. The loop brushes were photographed to measure width and length, with images captured at an angle perpendicular to the loop and analyzed using ImageJ (Version 1.52a). The longest horizontal and vertical lines encompassing the loop were recorded for width and length, respectively (Supplementary Information 4).

### Statistical analysis

Cell count results (brushing and respective negative control) were applied to a 1-sided Wilcoxon matched-pairs signed-rank test with a significance level of 5% to test for a statis- tically significant difference between brushing and not brushing. Brushing tests were paired with their respective negative control and grouped per loop material. The software used was GraphPad Prism 8 software (GraphPad, CA, USA).

## Results and discussion

No cyst perforations were observed during the study.

Loop brushes of Monocryl, PDS II, and Catgut demonstrated statistically significant (p-value = 0.039) efficacy in brushing cysts, while those of Vicryl, Chirasorb, Chirlac, or Glycolon did not (Fig. 3a). Nitinol brushes exhibited the highest brushing efficiency, averaging a factor 9.4 × . PDS II, Catgut, and Monocryl brushes yielded an average of 4, 2.4, and 3 times higher cell yields, respectively.

The measurements suggest a critical minimum flexural rigidity, between that of the Gly- colon and Nitinol brushes, for significant brushing (Fig. 3b). For loops with flexural rigidities between that of Nitinol and PDS II, efficiency appears to correlate with the width- to-length ratio of the loop. Nitinol loop brushes, which have moderate flexural rigidity but the largest width-to-length ratio, showed the highest brushing efficiency. In contrast, Catgut loop brushes, possessing the highest flexural rigidity but the smallest width-to-length ratio, exhibited only moderate brushing efficiency.

The mechanical properties of the loop material were identified as the primary factor affecting cell removal from cysts. We propose that brushing efficiency depends on the normal force between the brush and the cyst wall, as well as the brush’s ability to conform to the cyst wall. These factors are influenced by two mechanical properties: stiffness, indicated by the flexural rigidity, and plasticity, indicated by loop elongation after subsequent needle insertion and protrusion. Stiffness describes the level of mechanical interaction between a material and its surroundings. Materials with lower stiffness conform better to tissue topography, while those with higher stiffness exert greater mechanical force for a given deflection. The extent of plastic deformation of the loop during needle insertion determines the loop shape upon exiting the needle. Elongated loops, characterized by a low width-to-length ratio, result in reduced cyst area coverage during brushing.

We speculate that adjusting the size of the loop would address matching cyst size.

Absorbable cyst brushes could be readily integrated with the current praxis of FNA in clinical use. Medical doctors would need to learn how to operate the brush, but no other aspects of sample collection, preparation, or analysis would be modified.

Nitinol is the most efficient material for brushing cysts but is unabsorbable by the human body. We identify the PDS II brushes as the best absorbable brush, with Monocryl and Catgut brushes as possible alternatives.

Further experiments in living animals are required to evaluate brush efficiency in living tissue.

## Conclusions

In this study, we investigated the ability of seven absorbable loop brush materials to collect cells from man-made cysts during fine-needle aspiration procedures. PDS II loops were identified as the most effective, followed by Monocryl and Catgut loops. Brushing efficiency was found to depend on the normal force between the brush and the cyst wall, as well as the brush’s ability to conform to the cyst wall, which are influenced by the stiffness and plasticity of the loop material. Further *in vivo* studies in living animals are warranted to assess the performance of these absorbable loop materials in live tissue and to confirm their potential use in minimally invasive diagnostic procedures. By identifying suitable absorbable materials for loop brushes, this research contributes to the ongoing efforts to enhance the safety and efficacy of cell brushing during FNA, especially in hard-to-reach areas of the human body.

### Supplementary Information

Below is the link to the electronic supplementary material.

Supplementary file1 (PDF 253 KB)

## Data Availability

Data available within the article or its supplementary materials.
