# Supplementary material for: Absorbable cyst brushes
Source: Biomed Microdevices. 2023 Aug 23;25(3):33. doi: 10.1007/s10544-023-00674-y (PMC10447279; doi:10.1007/s10544-023-00674-y)
Supplement: Supplementary file 1 — Supplementary file1 (PDF 253 KB) [file 10544_2023_674_MOESM1_ESM.pdf]

Appendix

Title:

Absorbable cyst brushes

Authors:
Filipe Marques,† Wouter van der Wijngaart,†
Niclas Roxhed∗,†,⊥

†KTH Royal Institute of Technology, Micro and Nanosystems, Malvinas v ̈ag 10, 100 44 Stockholm, Sweden
⊥MedTechLabs, Bioclinicum, Karolinska University Hospital, Solna, Sweden

Correspondence to:
Niclas Roxhed (roxhed@kth.se)

Table of contents

1. Artificial cyst
2. Cell counting results
3. Flexural rigidity setup and results
4. Width-to-length results

1. Artificial cyst

Prior to brushing experiments, cysts would be placed in a petri dish and filled with water. An example of cyst immediately before a brushing experiment can be seen in Figure 1.

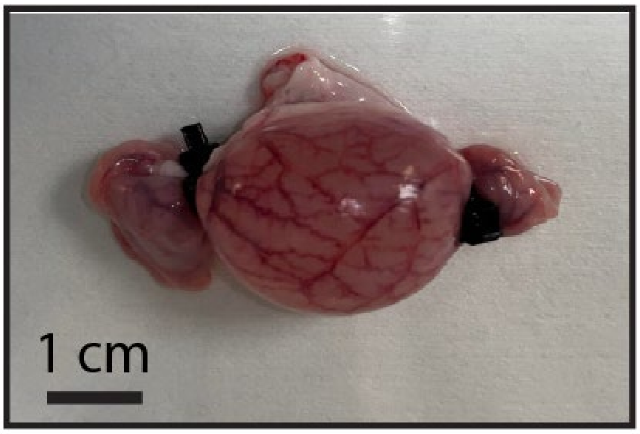

Figure 1 Photograph of an artificial cyst pre-testing produced from a small intestine of a pig. Cyst is filled with water.

2. Cell counting results

Table 1 Cell counting results (cells/ mL) relative to negative control and brushing of artificial cysts with Chirlac, Chirasorb, PDS II, Monocryl and Vycril loop brushes

# Experiment	Chirlac N	Chirlac	Chirasorb N	Chirasorb	PDS II N	PDS II	Monocryl N	Monocryl	Vicryl N	Vicryl
1	1870000	1690000	3730000	5030000	2210000	10300000	1550000	3660000	4410000	3410000
2	1950000	630000	4720000	4910000	3210000	12500000	1510000	3840000	3680000	6580000
3	1340000	548000	4640000	4600000	3280000	11800000	1350000	3240000	2650000	1110000
4	803000	1720000	988000	501000	214000	1230000	862000	1680000	545000	1060000
5	522000	748000	1260000	1810000	578000	1180000	129000	413000	1770000	956000
6	540000	560000	581000	589000	499000	1370000	1570000	2180000	358000	906000
7	968000	563000	622000	1050000	384000	1290000	1470000	1930000	994000	525000
8	707000	859000	1170000	208000	323000	1800000	519000	2120000	759000	1280000

Table 2 Cell counting results (cells/ mL) relative to negative control and brushing of artificial cysts with Glycolon, Catgut and Nitinol loop brushes

# Experiment	Glycolon N	Glycolon	Catgut N	Catgut	Nitinol N	Nitinol
1	5620000	2790000	384000	2050000	2230000	11000000
2	3300000	1510000	522000	3160000	132000	3570000
3	1740000	2230000	551000	1460000	-	-
4	138000	267000	93800	108000	-	-
5	411000	789000	70400	150000	-	-
6	1070000	1130000	499000	801000	-	-
7	396000	408000	405000	1040000	-	-
8	707000	531000	167000	452000	-	-

3. Flexural rigidity results

Table 3 Flexural rigidity measurements (mg).

# Experiment	Chirlac	Chirasorb	PDS II	Monocryl	Vicryl	Glycolon	Catgut	Nitinol
**1**	31	10	323	321	163	286	672	300
**2**	33	14	332	330	151	276	665	306
**3**	36	17	337	325	164	290	678	302

4. Width-to-length results

Table 4 Width (W) and Length (L) in pixels for all loop brush materials after protrusion from a 22G needle.

	Chirlac		Chirasorb		PDS II		Monocryl		Vicryl		Glycolon		Catgut		Nitinol	
# Loop	W	L	W	L	W	L	W	L	W	L	W	L	W	L	W	L
1	30	103	40	114	46	110	37	116	47	95	35	89	11	85	70	100
2	26	110	50	118	35	111	33	119	60	107	42	99	17	100	66	95
3	28	104	55	121	41	90	35	110	55	105	47	101	12	104	65	108

Table 5 Width-to-Length ratio for all loop brush materials after protrusion from a 22G needle.

W/L	Chirlac	Chirasorb	PDS II	Monocryl	Vicryl	Glycolon	Catgut	Nitinol
1	0,27957	0,350877	0,418182	0,318966	0,494737	0,393258	0,124706	0,7
2	0,236842	0,423729	0,315315	0,277311	0,560748	0,424242	0,17	0,694737
3	0,266667	0,454545	0,455556	0,318182	0,52381	0,465347	0,115385	0,601852
